# B Cell Depletion Reduces the Number of Autoreactive T Helper Cells and Prevents Glucose-6-Phosphate Isomerase-Induced Arthritis

**DOI:** 10.1371/journal.pone.0024718

**Published:** 2011-09-08

**Authors:** Oliver Frey, Lisa Bruns, Lars Morawietz, Kyri Dunussi-Joannopoulos, Thomas Kamradt

**Affiliations:** 1 Institute of Immunology, Jena University Hospital, Jena, Germany; 2 Institute of Clinical Chemistry and Laboratory Diagnostics, Jena University Hospital, Jena, Germany; 3 Institut of Pathology, Charité University Hospital Berlin, Berlin, Germany; 4 Pfizer Research, Cambridge, Massachusetts, United States of America; University of Muenster, Germany

## Abstract

The therapeutic benefit of B cell depletion in patients with rheumatoid arthritis has provided proof of concept that B cells are relevant for the pathogenesis of arthritis. It remains unknown which B cell effector functions contribute to the induction or chronification of arthritis. We studied the clinical and immunological effects of B cell depletion in glucose-6-phosphate isomerase-induced arthritis. We targeted CD22 to deplete B cells. Mice were depleted of B cells before or after immunization with glucose-6-phosphate isomerase (G6PI). The clinical and histological effects were studied. G6PI-specific antibody responses were measured by ELISA. G6PI-specific T helper (Th) cell responses were assayed by polychromatic flow cytometry. B cell depletion prior to G6PI-immunization prevented arthritis. B cell depletion after immunization ameliorated arthritis, whereas B cell depletion in arthritic mice was ineffective. Transfer of antibodies from arthritic mice into B cell depleted recipients did not reconstitute arthritis. B cell depleted mice harbored much fewer G6PI-specific Th cells than control animals. B cell depletion prevents but does not cure G6PI-induced arthritis. Arthritis prevention upon B cell depletion is associated with a drastic reduction in the number of G6PI-specific effector Th cells.

## Introduction

The therapeutic success of the B cell–depleting drug Rituximab (anti-CD20 antibody) in treating patients with rheumatoid arthritis (RA) has induced intensified interest in how B cells contribute to the pathogenesis of autoimmune diseases. B cells can produce autoantibodies and function as antigen-presenting cells (APC), which can profoundly influence T-helper (Th) cell proliferation and effector functions. They produce cytokines and regulate lymphoid tissue architecture and neogenesis. Which of these functions are relevant for RA and how B cell depletion exerts its therapeutic effect is currently unclear.

The identification of the mechanism by which B cells contribute to the induction or propagation of chronic synovitis in RA is severely hampered by the fact that analysis of the effects of B cell depletion is often restricted to peripheral blood, which contains only a minority of the total B cell population (less than 2%, [1]). For mechanistic studies, preclinical arthritis models are clearly needed. B cell depletion studies have been performed in collagen-induced arthritis (CIA) [2], [3], proteoglycan-induced arthritis [4] and arthritic K/BxN mice [5]. We chose to examine the effect of B cell depletion in glucose-6-phosphate isomerase (G6PI)-induced arthritis. Upon one single immunization with G6PI in adjuvant, peripheral symmetric polyarthritis develops with high incidence in genetically susceptible strains of mice [6], [7]. G6PI is also the target autoantigen in the transgenic K/BxN mice, which develop high titers of anti-G6PI specific autoantibodies [8], [9]. These autoantibodies are arthritogenic in the K/BxN transfer model, where transfer of serum or G6PI-specific mAbs generated from the transgenic K/BxN mice is sufficient to induce the disease in recipient mice [8], [10]. In arthritis induced by immunization of non-transgenic mice with G6PI in CFA, the role of B cells and/or autoantibodies is much less clear. We have shown that mice lacking mature B cells are fully resistant against G6PI-induced arthritis [7]; in mice lacking the FcγR common gamma chain G6PI-induced arthritis occurs at a very low incidence and strongly reduced severity, whereas G6PI-induced arthritis is more severe and chronic in mice lacking the inhibitory FcγRIIB [6]. However, in contrast to the K/BxN model and collagen-induced arthritis (CIA), G6PI-induced arthritis can not be transferred into syngeneic recipients by adoptive transfer of serum from diseased animals [6]. Thus, B cells and antibodies are necessary but not sufficient for the pathogenesis of G6PI-induced arthritis.

To deplete B cells we targeted CD22, a lectin-like member of the Ig superfamily, which is expressed exclusively by all mature B cells [11]. Antibodies targeting CD22 are available for human use as well, and are currently investigated in clinical trials. To target CD22^+^ cells we used an anti-CD22 monoclonal antibody conjugated with calicheamicin (referred here as CD22-cal) that efficiently depletes mature B cells in mice [2]. CD22-cal has been extensively characterized and used in animal models of arthritis, infection and type 1 diabetes [2], [12].

## Materials and Methods

### Mice induction of arthritis and treatment

Female SJL/J mice were bred and maintained in our specific-pathogen free animal facility. All experiments were approved by the appropriate governmental authority (Thüringer Landesamt für Lebensmittelsicherheit und Verbraucherschutz; registration numbers 02-005/06 and 02-024/04) and conducted in accordance with institutional and state guidelines. Recombinant human G6PI was prepared as previously described [6]. Six- to 12-wk-old mice were immunized subcutaneously at the base of the tail with 400 µg G6PI emulsified in 200 µl CFA (Sigma-Aldrich, Taufkirchen, Germany). Animals were scored for clinical signs of arthritis (erythema, swelling, ankylosis) on a 0–3 point scale for each paw, giving a total maximum score of 12. Histopathological assessment was performed as described before [6] by a pathologist (L.M.) who was blinded with respect to the experimental groups. For depletion of B cells, mice were treated with an immunotoxin consisting of anti-mouse CD22 mAb (Cy34.1) conjugated to *N*-acetyl-γ-calicheamicin dimethyl acid (CD22-cal) as described [2]. At the indicated time points mice were intraperitoneally injected with 150 µg CD22-cal (containing ∼3 µg calicheamicin) per injection. For serum transfer studies, 250 µl pooled sera from either arthritic (d15–20 after immunization) or naïve mice were injected intraperitoneally at days 9 and 11 after immunization.

### Anti-G6PI-Ig ELISA

The amount of G6PI-specific antibodies were measured by ELISA in serially diluted serum samples as previously described [6].

### Flow cytometry

For analysis of B cell depletion we isolated lymph nodes, spleens, peripheral blood and bone marrow (from femurs) at the indicated time points. After preparation of a single cell suspension and lysis of erythrocytes, cells were stained with fluorochrome-conjugated anti-bodies against CD3, CD11b and CD19 for 20 minutes on ice. After washing, cells were acquired using a BD LSRII flow cytometer. Data were analyzed using FlowJo 8.1.1 (Treestar Inc., Ashland, Oregon).

For analysis of G6PI-specific T cells, cells from draining lymph nodes (inguinal, axillary, paraaortic; 1×10^7^/ml) were cultured for 6 hours with 20 µg/ml G6PI or left unstimulated. For the last 4 hours, Brefeldin A (Sigma) at 5 µg/ml was added to all samples. At the end of the restimulation period, cells were washed and incubated with the fixable amine-reactive Aqua viability stain (Invitrogen) for 30 minutes on ice, fixed with 2% paraformaldehyd in PBS and permeabilized with 0.5% Saponin/0.5% BSA/0.02%NaN_3_ in PBS. Non-specific binding of antibodies was blocked by preincubation of the cells with anti-CD16/32 (2.4G2) and rat IgG (both at 5 µg/ml) for 8 minutes, followed by staining with fluorochrome-conjugated monoclonal antibodies against CD4, CD154, TNF-α, RANKL, IL-2, IL-17 and IFN-γ (all from eBiosciences or Miltenyi Biotech). At least 2.5 million events were acquired. Gates for CD154 were set using unstimulated control samples and gates for cytokine+ cells were set using fluorescence-minus-one controls for the respective cytokine.

For supplementation of antigen-presenting cells, single cell suspension of splenocytes from immunized mice were labeled with CFSE (5 µM for 3 minutes in ice). After washing with PBS, cells were adjusted to 1×10^7^/ml and mixed in a 1∶1 ratio with the lymph node cells of either control or anti-CD22-cal treated mice. This mixture was pulsed with antigen, stained and analyzed exactly as described above.

### Statistics

Data were analyzed using the non-parametric Man-Whitney U test with SPSS 15.0 (SPSS Inc., Chicago, USA) unless otherwise indicated. Graphs were generated using SigmaPlot 10.0 (Systat Software Inc, Chicago, USA). Data is presented as means ± standard error of mean (SEM) unless otherwise indicated.

## Results

### SJL mice are susceptible to G6PI-induced arthritis

To investigate the role of B cells in G6PI-induced arthritis at different disease stages, we wished to use a B cell depleting immunoconjugate (anti-CD22 mAb conjugated to chalicheamicin, referred hereafter to as CD22-cal). G6PI-induced arthritis is usually induced in DBA/1 mice [6], [7] which do not express the Lyb-8.2 alloantigen [13]. The Cy34.1 antibody used in these studies reacts with CD22 in strains expressing the Lyb-8.2 alloantigen, such as C57BL/6 or SJL/J. We, therefore, examined SJL/J mice for their susceptibility to G6PI-induced arthritis.

**Figure 1:**
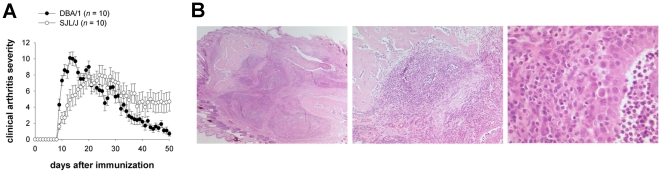
SJL/J mice are susceptible to G6PI-induced arthritis. A) Age matched female DBA/1 and SJL/J mice were immunized with 400 µg G6PI in complete Freund's adjuvant and scored for clinical arthritis severity as described in *Materials and Methods*. B) Histological features of G6PI-induced arthritis in SJL/J mice. The overview (left, ×25) shows the severe destruction of the joint. Cartilage is completely missing, and parts of the underlying bone are infiltrated as well (center, ×100). The inflammatory infiltrate consist mainly of neutrophil granulocytes, but lymphocytes and plasma cells are clearly visible (right, ×400). The underlying pannus consists of activated fibroblasts and is covered by an enlarged synovial lining. In the synovial space, a putrid exudate with numerous granulocytes is found (all figures, H&E staining).

Following immunization with one single dose of G6PI/CFA subcutaneously on day 0, SJL/J mice developed arthritis with the same cumulative incidence (more than 90%) and rapid onset as DBA/1 mice. Maximal clinical scores were reached somewhat later (> day 21) in SJL/J than in DBA/1 mice (< day 21) and G6PI-induced arthritis was more chronic in SJL/J compared to DBA/1 mice (Fig. 1A).

Clinical signs of arthritis were also confirmed by histology: Fig. 1B shows a very severe active arthritis with a dense infiltrate of neutrophil granulocytes, accompanied by lymphocytes, plasma cells, macrophages and activated fibroblasts in the synovial membrane. This tissue invades and destroys the joint cartilage and infiltrates deep into the underlying bone. The histological picture is similar to the infiltrates seen in DBA/1 mice, as previously described [6].

SJL/J mice generated G6PI-specific T cell- and antibody responses that were similar to those in DBA/1 mice regarding, quantity, quality and kinetics (data not shown).

### CD22-cal causes prolonged B cell depletion

To assess the extent and duration of B cell depletion in G6PI-immunized mice, SJL/J mice were treated with 150 µg CD22-cal at days −6 and −1 before immunization and immunized with G6PI at day 0. Spleens, lymph nodes, blood and bone marrow were collected at different time points after immunization and the frequency of CD19^+^ B cells and CD3^+^ T cells analyzed by flow cytometry. In peripheral blood of CD22-cal treated mice, CD19^+^ B cells were massively depleted over a long time. The anti-CD22-cal treated animals harbored only 4.62% (day 0); 6.71% (day 7); 27,1% (day 14); 17.86% (day 21) or 17.59% (day 28) of the peripheral blood B cells in control mice.

**Figure 2:**
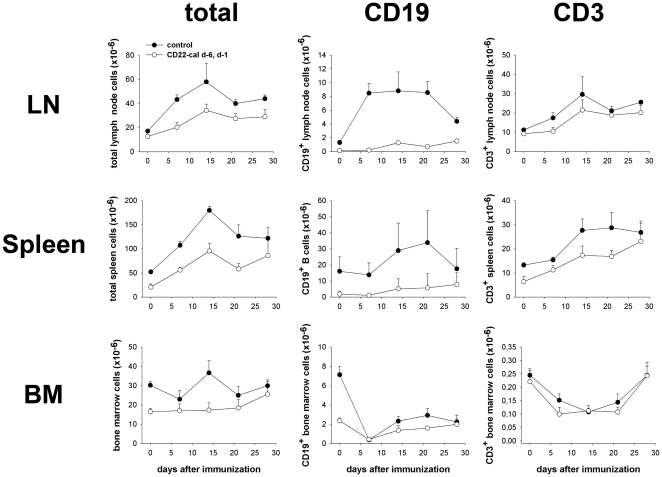
B cell depletion in CD22-cal treated mice. Mice were treated with CD22-cal at days −6 and −1 and were immunized with 400 µg G6PI in complete Freund's adjuvant. Control mice were immunized but otherwise left untreated. Lymph nodes (LN), spleens and bone marrow were isolated, counted and analyzed for the percentage of CD19^+^ B and CD3^+^ T lymphocytes. Shown are the total number cells harvested (left) or the total number of B- (middle) or T-lymphocytes (right) in the various organs. Data are representative for at least three mice per time point.

Lymph nodes and spleens in CD22-cal treated mice contained fewer cells compared to controls at all time points examined (between 47 and 74% of the controls; Fig. 2, left). The total number of bone marrow cells was also reduced in these mice. As expected, B cells were hardly detectable in the secondary lymphatic organs of CD22-cal treated mice within the first week after G6PI-immunisation, they started to recover slowly at approximately 2 weeks after the immunization but their numbers remained significantly lower than in control mice over the entire observation period. (Fig. 2, middle).

We also noted a slight decrease in the number of CD3^+^ T cells in spleens and lymph nodes of CD22-cal treated animals (Fig. 2, right). Although CD22-cal specifically depletes B cells, it is important to note here that the reduced B cell numbers do not fully explain the observed reduction in total cell numbers recovered from spleens and lymph nodes. For instance, the number of CD19^+^ B cells in lymph nodes from day 7 fell from ∼8.5×10^6^ to 0.2×10^6^ whereas the total cell number was reduced from 43×10^6^ to 20×10^6^. Taken together, our data show that treatment with CD22-cal leads to long lasting depletion of B cells in lymph nodes, spleen and blood and suggest that this B cell depletion indirectly affects the cellular composition of the secondary lymphoid organs in response to immunization as shown here by the reduced numbers of CD3^+^ T cells (Fig. 2, right).

### Prophylactic B cell depletion protects from arthritis induction

**Figure 3:**
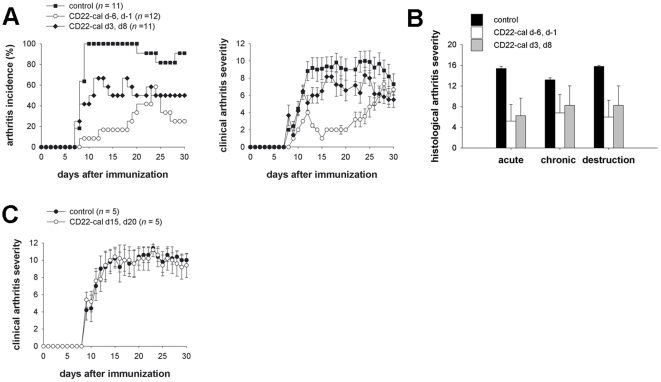
G6PI-induced arthritis in CD22-cal treated mice. SJL/J mice were immunized with 400 µg G6PI in complete Freund's adjuvant and treated at the indicated time points with CD22-cal. Mice were scored for clinical signs of arthritis. A) Treatment with CD22-cal before (day −6, day −1) immunization or in early preclinical stage (day 3, day 11) delay the onset and reduces the incidence of arthritis, compared to untreated controls (Left). Furthermore, prophylactic CD22-cal treatment strongly reduces the clinical severity of arthritis which still develop disease; this effect was much lower in mice treated in the induction phase (right). B) H&E stained sections were scored for the extend of acute (infiltration of neutrophil granulocytes) and chronic (infiltration of mononuclear cells) inflammation as well as joint destruction (cartilage and bone erosions) in a semiquantitative manner. For each parameter a maximum score of 4 could be reached. Shown are the sums score of all paws of individual at day 36 after immunization. C) Clinical score of mice that were treated with CD22-cal as described above at days 15 and 20 after immunization.

To assess the effect of B cell depletion on arthritis, mice received two injections of 150 µg CD22-cal either at days −6 and −1 or days 3 and 8 relative to immunization with G6PI. Mice treated prior to immunization showed a markedly delayed disease onset, the cumulative incidence reached only 50% and the clinical severity remained lower than in the control mice. When CD22-cal was injected at days 3 and 8 after immunization, arthritis incidence was still reduced to approximately 50% of controls (Fig. 3 A, left). Clinical arthritis scores of those mice that did develop disease after late CD22-cal treatment were lower than those of the control mice but higher than those of the mice, which had received prophylactic treatment (Fig. 3 A, right). We confirmed these findings by semiquantitative analysis of the histopathological changes (Fig. 3 B). Signs of acute and chronic inflammation as well as joint destruction were lower in either group of treated mice than in controls. We next asked if B cell depletion could also cure established G6PI-induced arthritis. As shown in Fig. 3 C, when the mice were treated at the peak of clinical disease (days 15 and 20 after immunization), anti-CD22-cal treatment was ineffective. Taken together, our data show that the critical role of B cells for pathogenesis of G6PI-induced arthritis is restricted to early phases of disease development.

### Lower anti-G6PI-specific Ig-levels in CD22-cal treated mice

**Figure 4:**
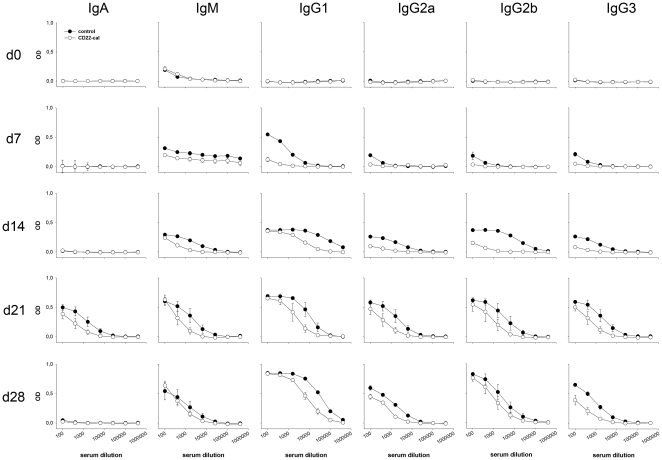
G6PI-specific Ig-levels in CD22-cal treated mice. Mice were treated with CD22-cal as in Fig. 2. Sera of mice were harvested at the indicated time points. Serially diluted sera (1/100, 1/400, 1/1600, 1/6.400, 1/25.600, 1/102.400, 1/409.600) were tested by ELISA for the presence of anti-G6PI-Ig's of the indicated isotypes. Shown are the optical densities, corrected for background. The data represent three indiviual mice per group and time point.

Antibodies against G6PI were almost absent in CD22-cal treated animals 7d after immunization (Fig. 4). At later time points (day 21 and 28), sera from CD22-cal treated mice contained anti-G6PI Igs of all isotypes, albeit at slightly lower levels compared to control animals (Fig. 4). Interestingly, the development of these titers occurred at a time point at which the numbers of B cells still were profoundly reduced (Fig. 2).

### Supplementation of G6PI-specific Ig does not restore arthritis development

**Figure 5:**
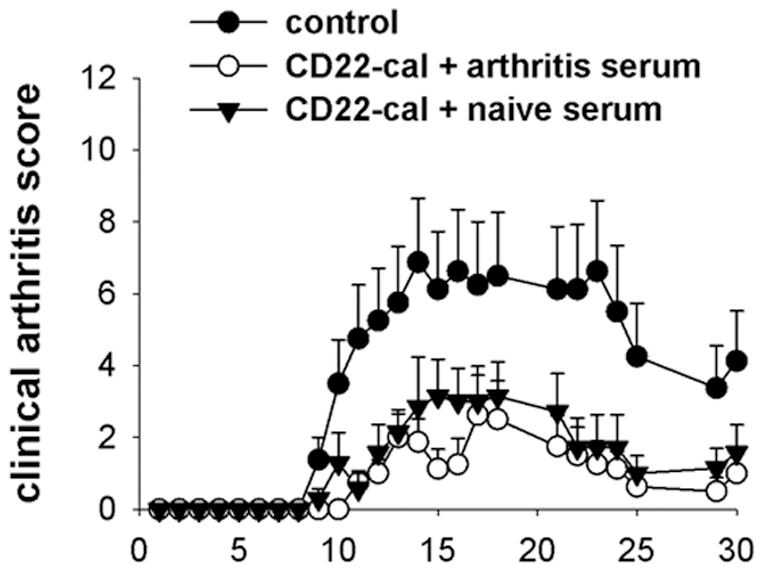
Serum transfer in CD22-cal treated mice. CD22-cal treated mice (day −6, day −1) or untreated mice were immunized with G6PI. At day 9 and 11, CD22-cal treated mice received 2 injections of pooled serum from either arthritic or naïve SJL/J mice. Shown is the clinical arthritis score from all mice per group (*n* = 8 per group).

Approximately 50% of the animals treated with CD22-cal at days -6 and -1 before immunization still developed arthritis, albeit with a delayed onset and reduced severity (Fig. 3 A). We hypothesized that this delayed disease induction might be due to the lack of G6PI-specific immunoglobulins at early time points. To test this we reconstituted B cell depleted mice with pooled sera from or naïve or arthritic mice at days 9 and 11 after immunization. Neither treatment could restore clinical arthritis scores in B cell depleted mice to the level in control mice. Furthermore, there was no difference between recipients of serum from arthritic or naïve mice (Fig. 5). These findings strongly argue against a lack of G6PI-specific immunoglobulins as the sole explanation for the beneficial effect of B cell depletion in G6PI-induced arthritis.

### Massively reduced G6PI-specific T cell response in B cell depleted mice

**Figure 6:**
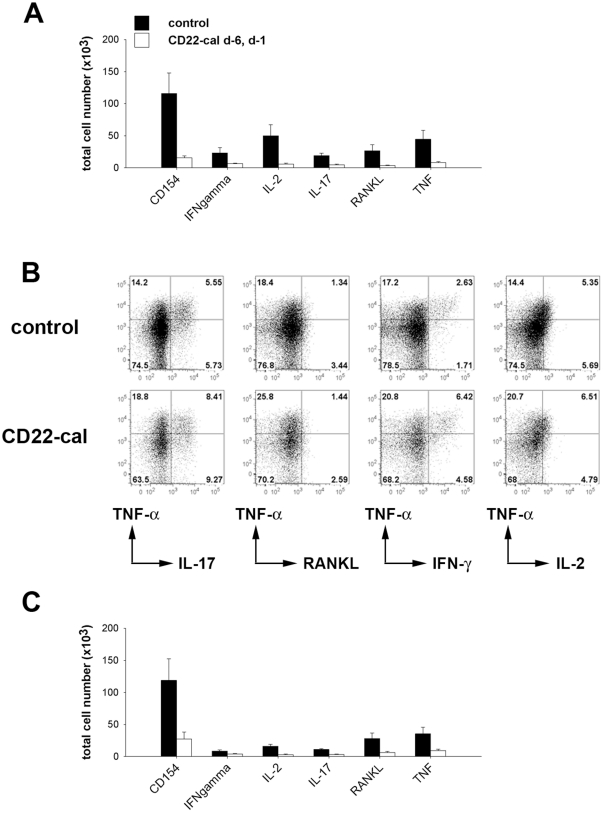
G6PI-specificTh cell response in CD22-cal treated mice. Mice were treated with CD22-cal at days −6 and −1 and were immunized with 400 µg G6PI in complete Freund's adjuvant. Control were immunized but otherwise left untreated. At day 9 after immunization, cells from the draining lymph nodes (inguinal, axillary, paraaortic) were restimulated with G6PI, fixed and stained for flow cytometry. A) Absolute number of G6PI-specific (CD154^+^) and G6PI-specific Th cells, expressing the indicated cytokine recovered from the draining lymph nodes of control or CD22-cal treated animals (*n* = 4 per group). B) Representative flow cytometry data from control (upper row) and CD22-cal treated (lower row) mice. Dot plots show expression of TNF-α (y-axis) against the other indicated cytokines by G6PI-specific (gated on CD154^+^) Th cells. The data shown are the concatenated data files from the 4 individual mice per group. C) Lymph node cells from the same mice as in A) and B) were stimulated in the presence of CFSE-labeled splenic antigen-presenting cells and similarly analyzed after gating on the CFSE-negative population.

Th cells are strictly required both in the induction- and the effector-phase of G6PI-induced arthritis [6]. We used multiparameter flow cytometry to compare the frequency and cytokine production of G6PI-specific T cells at day 9 after G6PI-immunization. Lymph node cells were briefly restimulated with G6PI, fixed and stained for CD154 and various cytokines for detection of antigen-specific T cells [14], [15], [16], [17]. Compared to the controls, the total number of CD154^+^ G6PI-specific Th cells was approximately 10-fold reduced in CD22-cal treated mice (11.6±3.2×10^4^ vs 1.6±0.3×10^4^; Fig. 6 A). This drastic reduction was due to the combination of fewer lymph node cells in CD22-cal treated animals (50.9±10.5×10^6^ in control vs. 20.4±2.8×10^6^ cells) and the reduced frequency of G6PI-specific cells within this reduced CD4^+^ cell pool (0.22±0.03 vs. 0.08±0.01% of CD4^+^ cells).

To assess the functional differentiation of the G6PI-specific Th cells, we next investigated the expression of the effector cytokines TNF-α, IL-17, RANKL, IFN-γ and IL-2. Among the G6PI-specific CD4^+^ T cells the proportions of cells producing RANKL- or IL-2 were similar in both groups of mice whereas the percentages of G6PI-specific CD4^+^ T cells that produced TNF-α, IL-17 or IFN-γ was higher in the CD22-cal treated mice than in the controls (Fig. 6 B). Approximately 27% of CD154^+^ cells from the treated mice expressed TNF-α, compared with only ∼20% CD154^+^ TNF-α producers in the control mice. Similarly, ∼18% of the G6PI-specific Th cells from the treated mice but only ∼11% from the control mice expressed IL-17 and for IFN-γ these percentages were ∼11 and ∼4, respectively (Fig. 6 B). Hence, although the size of the G6PI-specific T cell compartment was significantly reduced in CD22-cal-treated mice, within this compartment the proportion of cells producing effector cytokines was higher in the CD22-cal-treated mice than in controls. Together, the drastically reduced size of the G6PI-specific T cell compartment in combination with the higher frequency of cytokine producing cells within this compartment still resulted in a lower total number of cytokine secreting cells in CD22-cal treated mice (compare Figs. 6 A and 6 B).

### B-cell depletion results in impaired priming of G6PI-specific Th cells *in vivo*

Assays of antigen-specific Th cell responses depend on intact antigen-presentation during the brief *ex vivo* restimulation. Hence, it is possible that the reduced number of G6PI-specific Th cells response measured was not the consequence of disturbed *in vivo* priming, but rather due to disturbed antigen-presentation caused by a lack of B cells in our lymph node preparations from CD22-cal treated mice. To control for that, we added CFSE-labeled splenocytes from G6PI-immunized mice as additional APCs to the lymph node cells and restimulated this mixed culture with G6PI. CFSE-labeling of the splenocytes enabled us to identify them and to gate them out during FACS-analysis. When we analyzed these mixed cultures exactly as described above, we found that the added APCs were not able to increase the number of CD154^+^ or cytokine producing cells in CD22-cal treated mice (Fig. 6 C). This clearly demonstrates that the reduced number of G6PI-specific T cells in these mice was truly due to a *in vivo* priming defect and not to insufficient restimulation in the B cell depleted lymph node cultures.

## Discussion

Here report that *in vivo* depletion of B cells with CD22-cal prevents but does not cure G6PI-induced arthritis. The *ex vivo* correlates of protection were strongly reduced numbers of CD4^+^ G6PI-specific Th cells and reduced serum-concentrations of antibodies against G6PI.

Usually, DBA/1 mice are studied for G6PI-induced arthritis [6], [7], [14], [15], [18], [19]. The Cy34.1 antibody used in our current study reacts with CD22 on strains expressing the Lyb-8.2 alloantigen, which is not expressed in DBA/1 mice [13]. Therefore we examined if Lyb-8.2^+^ SJL/J mice were susceptible to G6PI-induced arthritis. The incidence and severity of G6PI-induced arthritis are similar in DBA/1 and SJL/J mice. Clinical signs of arthritis appear slightly later in SJL/J than in DBA/1 mice and G6PI-arthritis takes a more prolonged course in SJL/J mice. Thus, we here demonstrate for the first time the susceptibility of SJL/J mice for G6PI-induced arthritis.

Our finding that B cell depletion prevents G6PI-induced arthritis when performed before immunization, ameliorates arthritis when performed during the induction phase of the disease and has no therapeutic effect when performed in already arthritic mice extends earlier findings in other arthritis models. B cell depletion using CD22-cal in *ifng* ^−/−^ C57BL/6 mice prevented CIA [2]. Similarly, B cell depletion with an anti-murine CD20 mAb prevented CIA in DBA/1 mice but did not cure arthritic mice [3]. B cell depletion with an anti-murine CD20 mAb reduced only the severity but not the incidence of proteoglycan-induced arthritis in BALB/c mice [4]. In contrast, B cell depletion with the anti-human CD20 mAb Rituximab had no effect on arthritis in hCD20tg K/BxN mice. Rituximab was administered when mice were already arthritic and serum anti-G6PI IgG titers have reached maximal levels [5]. Finally, transfer of splenocytes from G6PI-immunized DBA/1 mice into SCID mice induced arthritis the recipients whereas transfer of CD19^+^-depleteds splenocytes did not [18]. Taken together, the data obtained from different mouse models of arthritis, using different strains of mice and different approaches to B cell depletion uniformly show preventive but not therapeutic efficacy.

These findings are in striking contrast to human RA where B cell depletion with Rituximab produces long-lasting robust clinical responses in many patients. Rituximab treatment does not affect long-lived plasma cells, yet the serum concentrations of rheumatoid factors are reduced upon Rituximab treatment [20]. Perhaps repeated T cell priming by B cells is necessary in RA but not in the murine models of arthritis, which are induced upon immunization with self-antigen in strong adjuvants?

B cells are important APC for CD4^+^ Th cells, which are critical for both the induction and the effector phase of G6PI-induced arthritis [6]. Therefore, we analyzed the G6PI-specific Th cell response in B cell depleted mice. T cell numbers were reduced in the draining lymph nodes of CD22-cal treated mice after G6PI-immunization although CD22-cal does not have a direct effect on T cells [2]. CD4^+^ T cells upregulate expression of CD154 rapidly and transiently upon TCR stimulation. CD154 expression is therefore highly indicative of antigen-specific CD4 T cell stimulation during the 6 h of in vitro culture [14], [16], [21], [22], [23], [24]. We found a strikingly reduced number of G6PI-specific Th cells in B cell depleted mice. The numbers of Th cells that produced IFN-γ, IL-17, RANKL, or TNF-α were also drastically reduced. This deficiency could not be overcome by *in vitro* stimulation with APC from syngenic mice that had not been treated with CD22-cal. Thus, we show here for the first time that T cell priming *in vivo* in the absence of B cells profoundly reduces the number of autoantigen-specific effector Th cells. This is supported by findings in the PGIA model where CD4^+^ T cells from B cell-depleted mice could not transfer disease into SCID recipients [4]. T cell priming seems to depend on antigen presentation by B cells especially when the dose of antigen is low [3]. The prominent role of B cells as APCs especially for low-abundant antigens might be explained by the fact that B cells very efficiently take up and present antigens at much lower concentrations than other APCs that sample antigens nonspecifically [3], [25]. Autoantigens such as CII, PG or G6PI are present only in low levels under normal circumstances. Thus, it is possible that after initial DC-mediated triggering of an autoantigen-specific Th response induced by immunization, the evolving B cell response might become more and more important for subsequent expansion and propagation of the specific Th cell response. Once the T cell response is established, B cells become less important which could explain the relatively small window of opportunity for the protective effect of B cell depletion. In this scenario, the reduced levels of autoantibodies could be interpreted as just an indicator of this reduced T/B cell cooperation without any significance for pathogenesis. The latter conclusion is, however, unlikely given the therapeutic effect of in vivo cleavage of IgG2a in CIA [26] or well-characterized protective effects of Fcγ-receptor blockade or complement deficiency [27], [28], [29], [30]. We have previously described a reduced severity of G6PI-induced arthritis in mice deficient for the Fcγ-receptor common γ chain and an exacerbated disease in mice lacking the inhibitory FcγRIIB [6], indicating the necessity for anti-G6PI Igs in the pathogenesis of G6PI-induced arthritis.

### Conclusions

In summary, our data show that B cell depletion is preventively but not therapeutically effective in G6PI-induced arthritis. Clinical signs of arthritis occur when B cells reappear in the blood and antibody-titers reach levels similar to those in control mice. B cell depletion results in a drastically reduced number of G6PI-specific Th cells. Thus, B cells are critical for the pathogenesis of G6PI-induced arthritis both as producers of antibodies and as APC for the pathogenic Th cells.

## References

[1] Westermann J, Pabst R (1990). Lymphocyte subsets in the blood: a diagnostic window on the lymphoid system?. Immunol Today.

[2] Dunussi-Joannopoulos K, Hancock GE, Kunz A, Hegen M, Zhou XX (2005). B-cell depletion inhibits arthritis in a collagen-induced arthritis (CIA) model, but does not adversely affect humoral responses in a respiratory syncytial virus (RSV) vaccination model.. Blood.

[3] Bouaziz JD, Yanaba K, Venturi GM, Wang Y, Tisch RM (2007). Therapeutic B cell depletion impairs adaptive and autoreactive CD4+ T cell activation in mice.. Proc Natl Acad Sci U S A.

[4] Hamel K, Doodes P, Cao Y, Wang Y, Martinson J (2008). Suppression of proteoglycan-induced arthritis by anti-CD20 B Cell depletion therapy is mediated by reduction in autoantibodies and CD4+ T cell reactivity.. J Immunol.

[5] Huang H, Benoist C, Mathis D (2010). Rituximab specifically depletes short-lived autoreactive plasma cells in a mouse model of inflammatory arthritis.. Proc Natl Acad Sci U S A.

[6] Schubert D, Maier B, Morawietz L, Krenn V, Kamradt T (2004). Immunization with glucose-6-phosphate isomerase induces T cell-dependent peripheral polyarthritis in genetically unaltered mice.. J Immunol.

[7] Bockermann R, Schubert D, Kamradt T, Holmdahl R (2005). Induction of a B-cell-dependent chronic arthritis with glucose-6-phosphate isomerase.. Arthritis Res Ther.

[8] Matsumoto I, Maccioni M, Lee DM, Maurice M, Simmons B (2002). How antibodies to a ubiquitous cytoplasmic enzyme may provoke joint-specific autoimmune disease.. Nat Immunol.

[9] Matsumoto I, Staub A, Benoist C, Mathis D (1999). Arthritis provoked by linked T and B cell recognition of a glycolytic enzyme.. Science.

[10] Maccioni M, Zeder-Lutz G, Huang H, Ebel C, Gerber P (2002). Arthritogenic monoclonal antibodies from K/BxN mice.. J Exp Med.

[11] Tedder TF, Poe JC, Haas KM (2005). CD22: a multifunctional receptor that regulates B lymphocyte survival and signal transduction.. Adv Immunol.

[12] Fiorina P, Vergani A, Dada S, Jurewicz M, Wong M (2008). Targeting CD22 reprograms B-cells and reverses autoimmune diabetes.. Diabetes.

[13] Symington FW, Subbarao B, Mosier DE, Sprent J (1982). Lyb-8.2: A new B cell antigen defined and characterized with a monoclonal antibody.. Immunogenetics.

[14] Frey O, Meisel J, Hutloff A, Bonhagen K, Bruns L (2010). Inducible costimulator (ICOS) blockade inhibits accumulation of polyfunctional T helper 1/T helper 17 cells and mitigates autoimmune arthritis.. Ann Rheum Dis.

[15] Bruns L, Frey O, Morawietz L, Landgraf C, Volkmer R (2009). Immunization with an immunodominant self-peptide derived from glucose-6-phosphate isomerase induces arthritis in DBA/1 mice.. Arthritis Res Ther.

[16] Frentsch M, Arbach O, Kirchhoff D, Moewes B, Worm M (2005). Direct access to CD4+ T cells specific for defined antigens according to CD154 expression.. Nat Med.

[17] Kirchhoff D, Frentsch M, Leclerk P, Bumann D, Rausch S (2007). Identification and isolation of murine antigen-reactive T cells according to CD154 expression.. Eur J Immunol.

[18] Tanaka-Watanabe Y, Matsumoto I, Iwanami K, Inoue A, Goto D (2009). B cells play a crucial role as antigen-presenting cells and collaborate with inflammatory cytokines in glucose-6-phosphate isomerase-induced arthritis.. Clin Exp Immunol.

[19] Iwanami K, Matsumoto I, Tanaka-Watanabe Y, Inoue A, Mihara M (2008). Crucial role of the interleukin-6/interleukin-17 cytokine axis in the induction of arthritis by glucose-6-phosphate isomerase.. Arthritis Rheum.

[20] Silverman GJ, Boyle DL (2008). Understanding the mechanistic basis in rheumatoid arthritis for clinical response to anti-CD20 therapy: the B-cell roadblock hypothesis.. Immunol Rev.

[21] Chattopadhyay PK, Yu J, Roederer M (2005). A live-cell assay to detect antigen-specific CD4+ T cells with diverse cytokine profiles.. Nat Med.

[22] Mittrucker HW, Steinhoff U, Kohler A, Krause M, Lazar D (2007). Poor correlation between BCG vaccination-induced T cell responses and protection against tuberculosis.. Proc Natl Acad Sci U S A.

[23] Gaucher D, Therrien R, Kettaf N, Angermann BR, Boucher G (2008). Yellow fever vaccine induces integrated multilineage and polyfunctional immune responses.. J Exp Med.

[24] Tokoyoda K, Zehentmeier S, Hegazy AN, Albrecht I, Grun JR (2009). Professional memory CD4+ T lymphocytes preferentially reside and rest in the bone marrow.. Immunity.

[25] Lanzavecchia A (1990). Receptor-mediated antigen uptake and its effect on antigen presentation to class II-restricted T lymphocytes.. Annu Rev Immunol.

[26] Nandakumar KS, Johansson BP, Bjorck L, Holmdahl R (2007). Blocking of experimental arthritis by cleavage of IgG antibodies in vivo.. Arthritis Rheum.

[27] Hietala MA, Jonsson IM, Tarkowski A, Kleinau S, Pekna M (2002). Complement deficiency ameliorates collagen-induced arthritis in mice.. J Immunol.

[28] Kleinau S, Martinsson P, Heyman B (2000). Induction and suppression of collagen-induced arthritis is dependent on distinct fcgamma receptors.. J Exp Med.

[29] Magnusson SE, Andren M, Nilsson KE, Sondermann P, Jacob U (2008). Amelioration of collagen-induced arthritis by human recombinant soluble FcgammaRIIb.. Clin Immunol.

[30] Diaz de Stahl T, Andren M, Martinsson P, Verbeek JS, Kleinau S (2002). Expression of FcgammaRIII is required for development of collagen-induced arthritis.. Eur J Immunol.

